# A new national smokefree law increased calls to a national quitline

**DOI:** 10.1186/1471-2458-7-75

**Published:** 2007-05-08

**Authors:** Nick Wilson, Gabriel Sertsou, Richard Edwards, George Thomson, Michele Grigg, Judy Li

**Affiliations:** 1 Department of Public Health, Otago University Wellington, PO Box 7343 Wellington South, New Zealand; 2 The Quit Group, PO Box 12 605, Wellington, New Zealand

## Abstract

**Background:**

A law making all indoor workplaces including bars and restaurants smokefree became operational in New Zealand in December 2004. New Zealand has a national free-phone Quitline Service which has been operational since 1999. Previous work has shown that the number of calls to the Quitline are influenced by marketing of the service through media campaigns. We set out to investigate if the smokefree law increased calls to the Quitline.

**Methods:**

For 24 months prior to the law, and 12 months after the law, data were collected on: (i) Quitline caller registrations and the issuing of nicotine replacement therapy (NRT) vouchers by the Quitline Service; (ii) expenditure on Quitline-related television advertising; (iii) expenditure on other smokefree television advertising; and (iv) print media coverage of smoking in major New Zealand newspapers. These data were inputs to a time series analysis using a Box-Jenkins transfer function model. This used the law change as the intervention variable, with the response series being the monthly Quitline caller rates and monthly first time NRT voucher issue rates.

**Results:**

The monthly rates of Quitline caller registrations and NRT voucher issues were observed to increase in the months after the law change. The increase in both these outcomes was even greater when considered in terms of per level of Quitline advertising expenditure (though these patterns may have partly reflected marked reductions in advertising expenditure at the time of the law change and hence are of limited validity).

In the more robust time series analyses, the law change (intervention variable) had a significant effect (p = 0.025) on increasing the monthly caller registration rate in December 2004. This was after adjusting for the possible effects of Quitline advertising expenditure, print media coverage, and other smoking-related advertising expenditure.

**Conclusion:**

The new national smokefree law resulted in increased quitting-related behaviour. This would suggest there is an extra opportunity for health agencies to promote quitting at such times.

## Background

The Smoke-free Environments Act passed in New Zealand in 1990 made many indoor workplaces smokefree, including: shops, most offices and some other workplaces (along with partial restrictions on smoking in cafés and restaurants). In December 2004, nearly all the provisions of the new *Smokefree Environments Amendment Act *of 2003 became operational. This Act had the effect of making all bars and restaurants completely smokefree, along with nearly all other workplaces and associated facilities not covered by the 1990 Act (eg, warehouses, factories and lunchrooms). The available evidence indicates that this new law has been well accepted by the public and has effectively improved air quality in settings such as bars and restaurants [1,2].

New Zealand has a national free-phone Quitline Service that is combined with the provision of nicotine replacement therapy (NRT) [3]. Calls to the Quitline are known to be increased by the level of advertising promoting smoking cessation that includes the Quitline telephone number [4,5].

A previous study has reported that there was a statistically significant increase in the number of new callers registering with the Quitline to make a quit attempt in the two months after the new law was implemented [6]. There was also a statistically significant increase in the dispensing of "exchange cards" for NRT (these are vouchers for obtaining heavily subsidised nicotine patches or gum from a pharmacy) over the same period. Week-by-week analyses also showed significantly increased caller registration rates in the week of the law change and in the subsequent week (even though it was the week before Christmas). However, these analyses were limited to analysing data over a two-month period (December-January) compared with the same period a year prior to the introduction of the new law, and did not take into account potential confounding factors such as the level of promotion of the Quitline service. In this article we examine a longer time period and take into consideration expenditure on different forms of media advertising and unpaid print media publicity on smoking.

## Methods

### Data sources

The Quitline routinely collects data on caller registrations and data on the distribution of NRT vouchers (and makes the information on the total number of calls and vouchers dispensed publicly available). These data were collated by month for 24 months prior to the law change and 12 months afterwards (Table 1). We also obtained national television advertising expenditure data by month from the agency that purchases television advertising time for the Quitline (Graham Strategic Ltd) (Table 1). Quitline advertising is focused around encouraging smoking cessation and a large majority of advertisements contain the Quitline number.

**Table 1:** Monthly Quitline caller data and advertising expenditure for 24 months before and 12 months after the new national smokefree law

**Year**	**Month**	**Registered callers (N)**	**First NRT vouchers issued from the Quitline (N)**	**Quitline advertising expenditure ($NZ 000s)**	**Other smoking related advertising expenditure ($NZ 000s)**	**Print media in major NZ news-papers (N)**
2002	December	1948	1069	2	13	36
2003	January	3436	1326	165	6	38
2003	February	3246	1610	307	37	34
2003	March	3389	1659	498	14	76
2003	April	3824	1656	198	23	44
2003	May	3555	1648	682	66	45
2003	June	3633	1603	330	39	34
2003	July	3686	1914	406	57	45
2003	August	3118	1494	781	0	62
2003	September	3022	1647	140	661	30
2003	October	2154	1098	98	574	54
2003	November	1783	900	7	1	37
2003	December	1353	751	0	0	52
2004	January	2592	1127	189	24	28
2004	February	2143	717	165	327	21
2004	March	3277	1672	161	0	31
2004	April	2585	1191	220	314	29
2004	May	3122	1161	128	759	29
2004	June	2772	1572	209	160	52
2004	July	2765	1292	223	383	25
2004	August	3115	1258	154	369	22
2004	September	2691	1329	0	414	30
2004	October	2549	1025	234	121	26
2004	November	2722	1698	140	591	57
2004	December*	2583	1642	1	209	115
2005	January	3130	1968	0	411	39
2005	February	2446	1181	45	367	45
2005	March	3306	1943	73	359	22
2005	April	2391	1539	0	522	52
2005	May	2263	1664	335	422	59
2005	June	1730	1095	160	225	32
2005	July	1948	1148	166	239	49
2005	August	2345	1473	156	281	41
2005	September	2329	1258	219	297	31
2005	October	2232	1162	177	0	38
2005	November	1840	965	0	0	29

* Month that the new law came into force.

To address other potential influences on calls to the Quitline we also collated national advertising expenditure data on other smokefree television advertising that covered themes other than smoking cessation, and which rarely included the Quitline number (Table 1). For example, there was a media campaign on not smoking in homes that was run from April 2004 by the Health Sponsorship Council (HSC) [7], and a media campaign on the forthcoming smokefree legislation that was run in late 2004 by the HSC [8]. Each year (in May) there was also a modest amount of "World Smokefree Day" television publicity.

Print media publicity is known to stimulate calls to the New Zealand Quitline^9 ^and so we collected monthly data on the number of articles covering smoking-related issues in major New Zealand newspapers from the "Factiva.com" Service (Table 1). The search term used in Factiva was "smoking or tobacco" in the category "major New Zealand newspapers" and for just within the "headline and lead paragraph" category.

### Statistical analysis

A Box-Jenkins transfer function model for time series was used with the analyses performed using SAS statistical software (Version 9.1, SAS Institute Inc, Cary, North Carolina, USA). Monthly caller rate and monthly first time NRT voucher issue rate between December 2002 and November 2005, were used as response series. The model input (explanatory) series were Quitline advertising expenditure, number of print media items, and other (non-Quitline) advertising expenditure over the same one-month periods. The law change effective from December 2004 onwards was the intervention variable.

Transfer function models were created using one of prewhitened monthly caller rate and monthly first time NRT voucher issue rate as the response series, the prewhitened explanatory series described above (in combination), and the intervention (in combination with prewhitened explanatory series) modelled in a variety of forms [9]. These were: an abrupt start and abrupt decay (impulse); an abrupt start and gradual decay; an abrupt start and permanent effect (step); and a gradual start and permanent effect. Residual sample cross-correlations, auto-correlations, and partial-autocorrelations for each model were checked to ensure statistical independence of error terms and validity of each model, as described by Bowerman [9].

## Results

**Figure 1:**
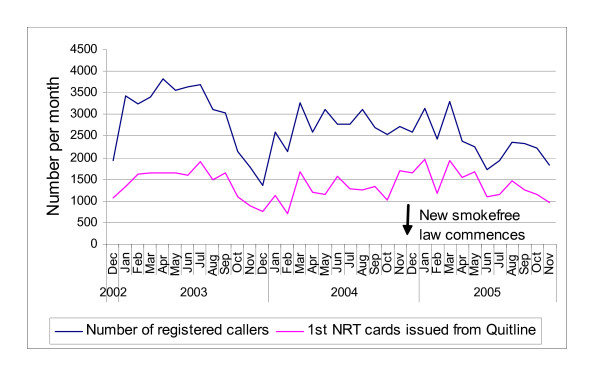
Monthly number of caller registrations with the Quitline and nicotine replacement therapy (NRT) exchange cards issued by the Quitline.

The monthly data (Figure 1, Table 1) suggest that the usual summer dip in both Quitline registrations and issues of NRT vouchers disappeared in December 2004/January 2005 when compared to the previous two years (the law change occurred in early December 2004). Indeed, caller registrations per month remained elevated (compared to the preceding year) for *every *month in the post-law change period through to the end of March 2005 and until the end of May 2005 for the issuing of the NRT vouchers. This pattern was despite a marked reduction in television advertising expenditure on promoting smoking cessation in early 2005 (see footnote to Table 2). The proportion of calls to the Quitline by Mâori callers in the six months after the law change was slightly lower than for the other five six-month periods (19.3% versus 20.2%) (Table 2). This lower proportion was only just statistically significant (rate ratio (RR) = 0.95; 95%CI = 0.92 – 0.99).

**Table 2:** Summarised Quitline caller and advertising expenditure data by six-month period for 24 months before and 12 months after the new national smokefree law

**Six-month time period**	**Registered callers (N)**	Call rate per 100,000 smokers**	**Callers who were Mâori (%)**	**First NRT vouchers issued from the Quitline (N)**	Issue rate per 100,000 smokers**	**Quitline advertising expenditure **($NZ 000s)	**Caller registrations per $1000 expend-iture**	**First NRT vouchers issued per $1000 expend-iture**
December 2002 to May 2003	19,398	2955	20.7	8968	1366	1852	10.5	4.8
June 2003 to November 2003	17,396	2650	22.3	8656	1319	1762	9.9	4.9
December 2003 to May 2004	15,072	2296	19.1	6619	1008	863	17.5	7.7
June 2004 to November 2004	16,614	2531	19.4	8174	1245	958	17.3	8.5
December 2004* to May 2005	16,119	2455	19.3	9937	1514	391***	41.2	25.4
June 2005 to November 2005	12,424	1892	19.6	7101	1082	943	13.2	7.5

* Month that the new law came into force.

** Rates are based on an estimated total population of smokers in New Zealand aged 18 years and over of 656,489 (based on rates from the 2002/2003 NZ Health Survey and 2004 population data estimates).

** The relatively low level of spending at this time was when the Quit Group were preparing to re-configure the Quitline Service (which started in early May 2005). This meant that there was a transitional period when the Quitline number was not included on advertising as staff were being trained in operational aspects of the new service. The re-configured Quitline Service involved discontinuing the use of an external call centre and having all incoming calls answered directly by the Quitline Advisors. In addition, follow-up support and advice (including mailed out information) became more customised to the caller's level of motivation for quitting smoking (based on a stage of change assessment).

The summarised data by six-month period indicate that Quitline caller registrations, per dollar of advertising directly linked to smoking cessation, showed at least a doubling in the six months after the law change, relative to the other six-month periods (Table 2). The same pattern was apparent for the issuing of NRT vouchers, with at least a three-fold higher level per advertising dollar compared to the other six-month periods. However, because the Quitline advertising expenditure declined at the time of the law change, these patterns for callers *per level of expenditure *may not entirely reflect law change-attributable demand for the Quitline service. Hence the time series analysis we undertook considered the monthly caller registration rate instead.

The time series analyses found that the law change intervention variable was only significant (p = 0.025) in the model with monthly caller rate as the response variable. The intervention was only significant in this model in the form of an impulse.

The model with monthly first time NRT voucher issue rate as the response variable, indicated no significant explanatory variables. The law change intervention included in this model came closest to significance (p = 0.073) when given an abrupt start and gradual decay form.

Neither the abrupt start and gradual decay, the step, nor the gradual start and permanent effect forms of the law change intervention reached significance (at the α = 5% level) in any of the models investigated.

## Discussion

The results show that there was a statistically significant increase in the rate of Quitline caller registrations in response to the law change. These results took into account other smokefree-related television advertising expenditure and print media publicity on smoking issues. Such findings indicate that smokers increase quitting behaviour when smokefree environments policies are introduced.

The findings for Quitline calls are consistent with the previous New Zealand work that examined such calls in the weeks and a two-month period after the new law [6]. They are also consistent with other data that suggests that call rates to the New Zealand Quitline are influenced by a range of factors that would be expected to promote quitting. These include television-based media campaigns [4,5], improved access to NRT [3], and media publicity around smoking hazards [10]. Conversely the call rate drops significantly when major international events distract smokers from quitting [11] and also seasonally over the December-January summer holiday period (Figure 1).

The results in this study for the Quitline Service are also consistent with the other data that may be associated with the impact of this smokefree law on smoker behaviour in New Zealand. These include evidence for a decline in youth smoking rates and a decline in "parental smoking" reported by school students between 2004 and 2005 [1]. Survey data also indicate that the proportion of smokers who reported that they smoked "more than normal" at bars, nightclubs, casinos and cafés declined substantially between 2004 and 2005 (a much steeper decline than between 2003 and 2004) [12].

These findings for New Zealand are also consistent with the available international literature. Smokefree workplace policies elsewhere have been shown to reduce social cues for smoking, decrease tobacco consumption, and increase quit rates. For example, one analysis of 19 studies of smokefree workplaces found that 18 reported declines in daily smoking rates and 17 reported declines in smoking prevalence [13]. A systematic review also concluded that "smoke-free workplaces not only protect non-smokers from the dangers of passive smoking, they also encourage smokers to quit or to reduce consumption" [14]. Another review concluded that "smokers who are employed in workplaces with smoking bans are likely to consume fewer cigarettes per day, are more likely to be considering quitting, and quit at an increased rate compared with smokers employed in workplaces with no or weaker policies" [15]. More recently published studies are consistent with the findings in these reviews [16,17]. There is also evidence from tobacco industry internal documents that reveal that this industry views smoking restrictions in public places as being one of the most important threats to cigarette consumption – as detailed by Siegel et al [18]. With regard to recent national level law changes, there is also supportive evidence for increased quitting behaviour from Ireland [19] and from Italy [20].

### Limitations

The analysis considered Quitline advertising expenditure as well as television advertising on other smokefree themes. However, it did not adjust for the fact that different smoking cessation advertisements used in New Zealand appear to have different effects on calls to the Quitline [4,5] and that there was variable use of the higher impact advertisements over the time period studied (but we did not have precise enough data to undertake advertisement weightings). Furthermore, some of the Quitline television advertising was occasionally used for general public awareness raising purposes as opposed to maximising calls to the Quitline. Expenditure data on various tobacco control promotional activities undertaken at the local level by District Health Boards (albeit considered to be fairly minor) was also not included in the analysis since such data are not readily available.

### Implications for future tobacco control policies

The quitting-related changes associated with this new law suggest there are opportunities for maximising the cost-effectiveness of smoking cessation advertising and use of unpaid media at such times, and for maximising the impact of major tobacco control interventions by concurrent intensification of the promotion and provision of smoking cessation services so that smokers' needs can be adequately met. This is especially so since the Quitline advertising levels are relatively modest (eg, compared with road safety mass media campaigns in New Zealand). Unfortunately, in New Zealand the opposite occurred, with promotion of the Quitline restricted in the period after the implementation of the smokefree legislation.

It is also important to ensure that the benefit from the increased stimulus to quit due to tobacco control interventions is equitable across all ethnic and socioeconomic groups, particularly in New Zealand where smoking is very unevenly distributed, with much higher smoking prevalence among Mâori, the indigenous people of New Zealand. There is therefore a need for campaigns and services to be orientated towards population groups with the highest needs.

These findings also suggest that there could be major increases in quitting behaviour if the introduction of such laws were part of an overall "intense impact" strategy. That is, there could be simultaneous tobacco price changes, large increases in smoking cessation support capacity, and improved access to all proven smoking cessation technologies (eg, subsidised access to other forms of NRT, bupropion and nortriptyline).

## Conclusion

The new national smokefree law resulted in increased quitting-related behaviour at a country-level. These findings are consistent with other New Zealand data relating to this law and with published international literature on the impact of smokefree environment policies. These findings suggest there is an extra opportunity for health agencies to promote quitting when such policies are introduced.

## Competing interests

Two of the authors (MG and JL) work for The Quit Group (a not-for-profit organisation that runs the Quitline). NW has previously undertaken contract work for The Quit Group (in 2004).

## Authors' contributions

Several of the authors (NW, RE, GT) were involved in designing the project (as part of a larger study to evaluate the impact of the new smokefree law). MG and JL organised the data extraction. GS and NW analysed the data. All the authors were involved re-drafting of the manuscript and have given final approval of the version to be published.

## Pre-publication history

The pre-publication history for this paper can be accessed here:


